# Devastating “DANA” Floods in Valencia: Insights on Resilience, Challenges, and Strategies Addressing Future Disasters

**DOI:** 10.3389/phrs.2025.1608297

**Published:** 2025-04-28

**Authors:** Jose M. Martin-Moreno, Eva Garcia-Lopez, Mariano Guerrero-Fernandez, Jose L. Alfonso-Sanchez, Paul Barach

**Affiliations:** ^1^ Department of Preventive Medicine and Public Health, University of Valencia, Valencia, Spain; ^2^ Research and Development Department, EpiDisease S.L., Valencia, Spain; ^3^ Facultad de Ciencias de la Salud, Universidad Católica San Antonio de Murcia (UCAM), Murcia, Spain; ^4^ College of Population Health, Thomas Jefferson University, Philadelphia, PA, United States

**Keywords:** floods, natural disasters, emergencies, resilience, community resources, public policy

## Abstract

**Background:**

The unprecedented DANA floods that struck Valencia, Spain on October 29, 2024, caused catastrophic human, economic, and ecological damages, resulting in 223 fatalities, the displacement of 15,000 residents, and long-term health and environmental consequences and incurred financial losses estimated over 50 billion euros. Vulnerabilities in urban planning, ambiguous command and control oversight, delayed warnings, and insufficient preparedness underscored systemic gaps in the flood preparedness and management.

**Analysis and Policy Options:**

Urgent actions, including stricter urban zoning laws, implementing comprehensive early warning systems, and fostering community-based disaster preparedness are needed to mitigate future risks. A One Health approach that addresses the interconnectedness of human, animal, and environmental health is critical. Investments in resilient infrastructure and communication strategies, including mobile alerts and public education campaigns, can significantly mitigate overall impacts. Moreover, prioritizing mental health interventions and long-term recovery plans are essential for fostering community resilience.

**Conclusion:**

The Valencia floods catastrophe is a painful reminder of the devastating potential of extreme weather and the critical importance of preparedness and clear governance underscore the pressing needs for integrated disaster management frameworks. An effective system of cooperation between local and national governments is essential to avoid the confusion and delays seen during this disaster. Strengthening prevention, preparedness, and response measures, and fostering political and community cohesion, can enable societies to build adaptive capacity to effectively tackle the increasing challenges of climate-driven extreme weather disasters. Future leaders must act with urgency and transparency to build trust and put citizens' safety above all else.

## Background

Floods are the most common climate hazard and leave devastating consequences. Between 2000 and 2019, more than 1.65 billion people were globally affected by floods, and more than 100,000 lives were lost [1]. Record-breaking rainfall and flash *floods* hit Valencia, Spain on 29 October 2024, caused hundreds of casualties, massive disruption, and economic losses. This catastrophic storm, called DANA (an acronym in Spanish for *Depresión Aislada en Niveles Altos*, an isolated high-impact depression), is a meteorological phenomenon characterized by extreme weather events, particularly in regions like the Mediterranean Sea basin. *DANAs* are known for triggering frequent lightning, large hail events and tornadoes. This event impacted nearly 1 million people, resulted in loss of 223 lives, displacement of 15,000 residents, and lasting epidemiological impacts. Three people still remain missing. Most of the fatalities were over the age of 70, highlighting the vulnerability of older adults to excess morbidity and mortality associated with extreme weather events, including heatwaves, wildfires, and hurricanes. Furthermore, the floods resulted in increased respiratory infections and vector-borne diseases. Public health authorities reported a spike in infectious diseases, including a 20% increase in waterborne illnesses such as gastroenteritis and leptospirosis cases, exacerbated by contaminated water supplies and disrupted sanitation systems [2]. This disaster left a trail of destruction, despair, and pressing questions about national and regional leadership, preparedness, adaptation efforts and community resilience to climate change and extreme weather conditions.

The event underscores the vulnerabilities of both natural and urban systems, as well as the converging health risks to human and animal populations, making it a compelling case for a *One Health* perspective. Adopting this integrated approach allows for a deeper understanding of the root causes, anticipating the cascading effects, and the need to develop resilient systems to safeguard the health of people, animals, and the environment in the face of extreme weather events exacerbated by climate change [3].

**FIGURE 1:**
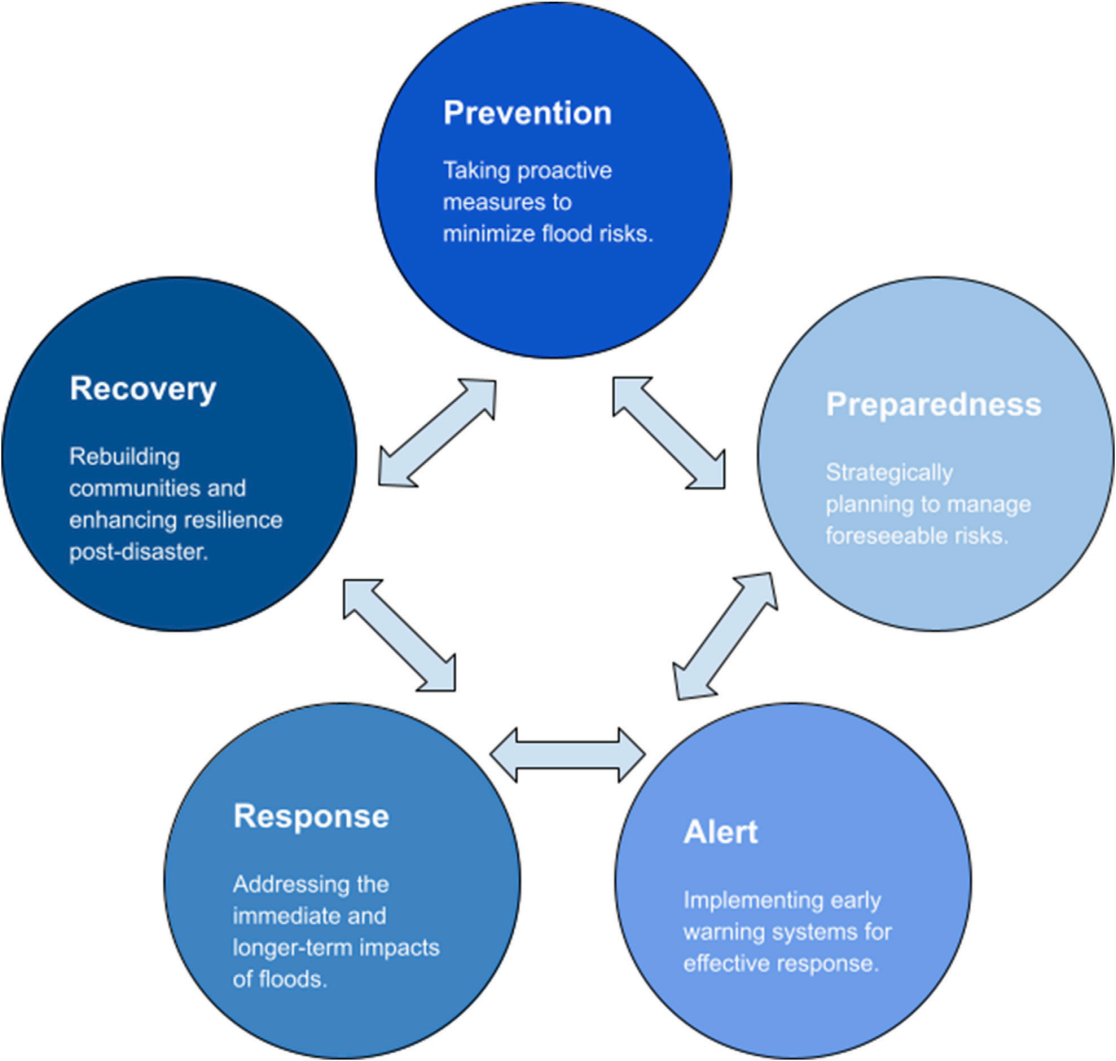
Key steps in flood disaster management: An integrated framework for comprehensive and resilient solutions. Valencia DANA Floods and Resilience (2024).

We examine the key aspects of this tragedy, by breaking down the key steps in disaster resilience (see Figure 1). We focus on the underlying issues of insufficient prevention and preparedness, weak regional leadership with significant delays in alerting the population, immediate response and recovery, and long-term political and community implications of the disaster. We draw on lessons from previous disasters to underscore the critical needs for rebuilding trust, and highlighting the political and social resolve needed in establishing resilient public health frameworks.

## Analysis and Policy Options

Our analysis and recommendations are grounded in a structured synthesis of the available scientific literature (with cited references our professional expertise and experience in the field, and case study evidence drawn from relevant public health practice.

### Prevention: Lessons From a Flood-Prone Area. What Could Have Been Done Better to Prepare for the Flood in Advance?

Unregulated construction in flood-prone areas, such as dry riverbeds (“ramblas”), amplified the damage [4]. Creating more effective building construction codes, including enforcing stricter zoning laws, can mitigate severe impacts. In the case of Valencia, high density urban areas made the flood hazard more dramatic and disastrous. Urban areas are especially vulnerable to natural hazards due to high population densities, limited opportunities for energy dissipation and the concentration of high-value assets [5]. The devastating floods in Germany in July 2021 exposed significant and incredibly similar gaps in community preparedness, resulting in over 200 fatalities and total estimated losses of €32 billion [6].

### Preparedness: Addressing Predictable Risks

Residents of flood prone zones should be aware of the underlying risks and be prepared for potential contingencies [7]. In the 2024 Valencia and Germany 2021 floods, civil preparedness including community education and training were missing. Education campaigns targeting all age groups are critical. Schools can effectively integrate emergency preparedness into their curricula [8]. For adults, preparedness drills have been shown to significantly enhance community resilience by increasing public understanding of flood-related risks and promoting appropriate self resilence, behavioral responses during floods.

### Alert: Failures With Early Warning Systems—Coupled to Actions to Mitigate Immediate Catastrophic Impacts

It is well known that effective early warning and dynamic risk management systems can save lives and reduce property damage [9]. Valencia had in place a citizen alert system that was suitable for raising public awareness and alerts. The system operated on two levels. First, the technical component was managed by the Spanish Meteorological Agency (AEMET), which issued informative warnings. Second, the warnings are the responsibility of the President of the Valencian Government (Generalitat Valenciana), who can activate the ES-Alert system directly to all registered citizens’ cell phones.

**FIGURE 2:**
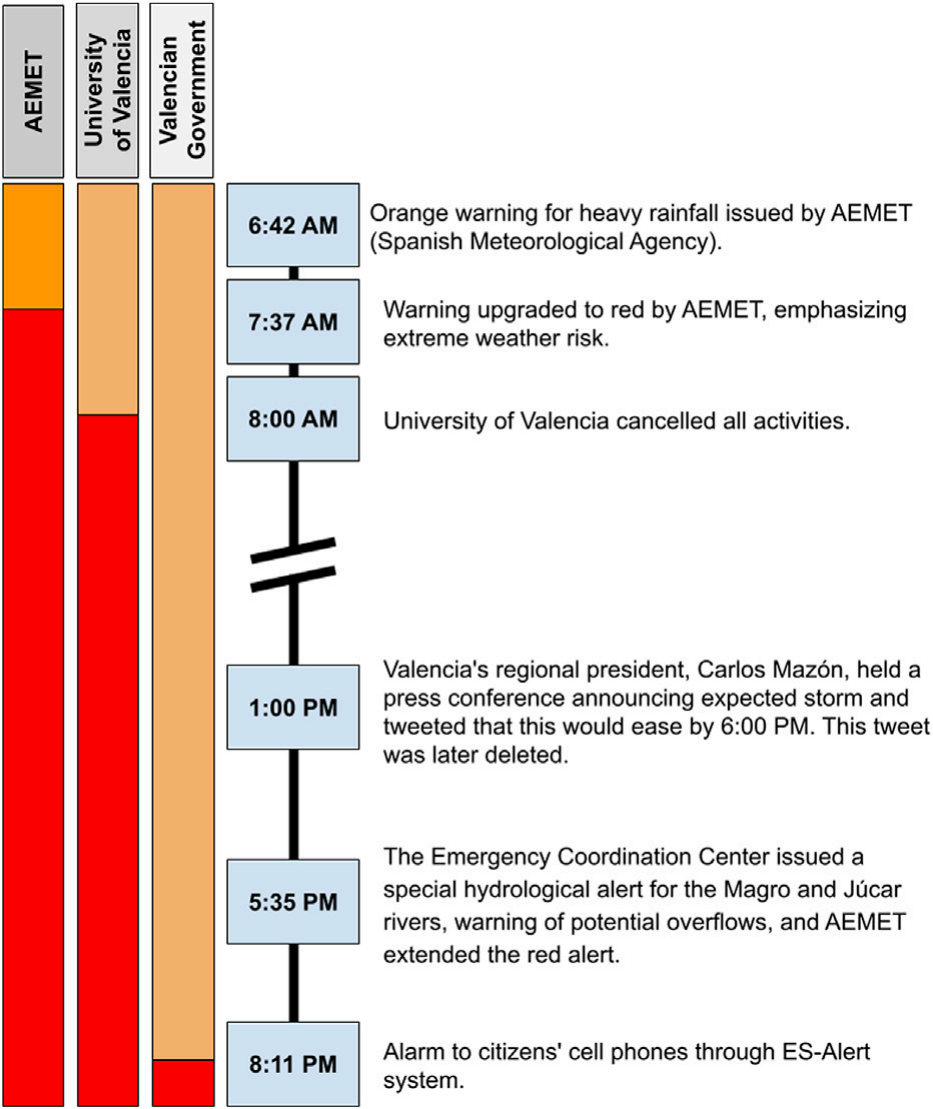
Timeline evolution of Warning and Alert Interventions during the Valencia Flood in Spain, 29 October 2024; The Spanish Metereological Agency’s traffic light system ranges between green (no meteorological risk), yellow (BE AWARE, stay informed of the most up-to-date weather forecast), orange (BE PREPARED, take precautions and stay informed of the most up-to-date weather forecast. Everyday outdoor activities may be affected), and red (take preventive measures and ACT according to the instructions of the authorities. Do not travel unless strictly necessary), with the latter being the most severe. The visualization shows only October 29, 2024, excluding green and yellow levels as they were issued on previous days. Valencia DANA Floods and Resilience (2024).

The sequence of warnings issued on 29 October 2024, is illustrated in Figure 2.

The following failures were identified in the alert management system:

• Delay in mobile alerts: Significant delays occurred between issuing meteorological warnings and the dissemination of alerts to community leaders and citizens, limited the population’s ability to take timely preventive and response measures.
• Underestimation of the phenomenon’s intensity: Initial forecasts, and the attention given to them, failed to align with the actual rate and intensity of rainfall and torrential water accumulation, adversely impacting preparedness and risk perception responses to the event. The main disaster risk factors of the 2021 Germany flood were seen to be related to late issuing and misunderstanding of warnings, a lack of information and data exchange, all aggravated further by critical infrastructure failures [10].
• Lack of inter-institutional coordination: Significant deficiencies in communication and coordination were perceived by citizens in flooded areas among national, regional, and autonomous authorities as well as emergency services, which undermined public trust and hindered the effectiveness of response efforts.

These failures are subjects of intense public debate, highlighting the need to improve alert and response protocols for meteorological emergencies and adequately protect the population in future events.

Disaster experience suggests two types of different responses to impending flood risks. On 28 October 2024, based on risk assessments informed by the data from the Spanish Meteorological Agency, the established protocols, and the experience accumulated in previous events, the University of Valencia proactively suspended all academic activities in anticipation of severe weather conditions [11] (see Figure 2). This decision was widely communicated to students and staff on the afternoon of 28 October following the activation of a level 2 (orange) emergency. And immediately after the AEMET declared a red alert early in the morning of 29 October, it was urged to avoid commuting/movements and all activities were cancelled. In contrast, the Generalitat Valenciana issued its public alert to citizens’ mobile phones only at 20:11 on 29 October several hours after the university’s announcement. The university’s timely response advising students and staff to stay home and avoid travel due to the hazardous conditions reduced significantly reduced risks and saved lives.

In general, more effective and decisive action could include:

• Proactive risk communication: Risk communication is multifaceted in various stages of the disaster life cycle. Timely and precise public alert warnings, leveraging mobile networks and media, could guide residents to safer and higher locations and discouraged risky actions, such as driving during the storm or attempting to rescue vehicles.
• Before the disaster, these systems can raise awareness of potential threats and encourage people to prepare and take mitigation actions. During a disaster it gives early and ongoing warnings and triggers a particular behavioural response. Interpretations and other subjective judgments about risks are known as risk perception. Risk communication requires gathering information and clearly understanding the current hazard situation. After a disaster, it can help in minimizing the damages of the hazard, such as through trusted and calm, evacuation messages and reliable route and shelter information.

Several studies have extensively discussed factors, theories, and frameworks of effective risk perception [12].

• Community preparedness: An effective Early Warning System (EWS) that integrates hazard monitoring, forecasting, risk assessment, communication, and preparedness can facilitate timely actions to mitigate disaster risks. Moreover, community-based alert systems and clearly marked evacuation routes have been shown to significantly reduce casualties and minimize confusion during disasters [13] However, in Valencia, these systems were not effectively utilized. The ES-Alert system, (Spain's public warning system) issued warnings too late—after the floods had already impacted over 25,000 ha and nearly 200,000 inhabitants.

### The Response at the Aftermath: Immediate and Long-Term Consequences

Extreme weather events influence human health and their communities. They can directly cause injuries or deaths and/or indirectly lead to physical illnesses, mental health disorders, damages to properties and infrastructures, water contaminations, as well as resurgence and redistribution of infectious diseases [14].

The infrastructure and homes in an area of over 300 square kilometers were severely damaged, with stagnant water creating a breeding ground for molds and waterborne diseases. The lack of adequate sanitation exacerbated these risks. The effects of the floods damaged and destroyed the sanitation infrastructure, disrupting services affecting a population of 200,000 inhabitants.

Traumatic injuries such as cuts, falls, injuries from flying or falling materials, and collisions with rapidly moving objects in floodwater were the main causes of injuries and fatalities during the flood. Additionally, chemicals stored in the environment were released leading to toxic water [15]. Floods are known to increase risks of communicable diseases and cutaneous and respiratory infections. Rodent-borne diseases, such as leptospirosis and hantavirus infection, were transmitted through direct contact with rodents or through contact with contaminated soil, food, or water [16].

The emotional toll around Valencia has been immense. Anguish, anxiety, post-traumatic stress, depression insomnia and other psychological disorders caused by the devastating floods in Valencia have inflicted deep-seated damages to the resident mental health [17]. In fact, it is well established that climate change impacts mental health through direct and indirect pathways [18]. Direct pathways include exposure to traumatic events, such as bushfires and other severe weather-related events. Indirect pathways include social, political, and economic determinants of mental health such as poverty, unemployment, and housing.

A third dimension derived from the environmental consequences can have a long-lasting impact on health and wellness, including:

- Water contamination. Floodwaters often contaminate water sources with industrial waste, pesticides, and sewage, reducing access to clean drinking water and causing long-term health issues.
- Ecosystem Degradation: The destruction of natural and agricultural areas leads to biodiversity loss, affecting the environment and local livelihoods.
- Impact on Food Security: The destruction of farmland and livestock diminishes food availability, increasing the risk of food insecurity in affected communities [19].

**FIGURE 3:**
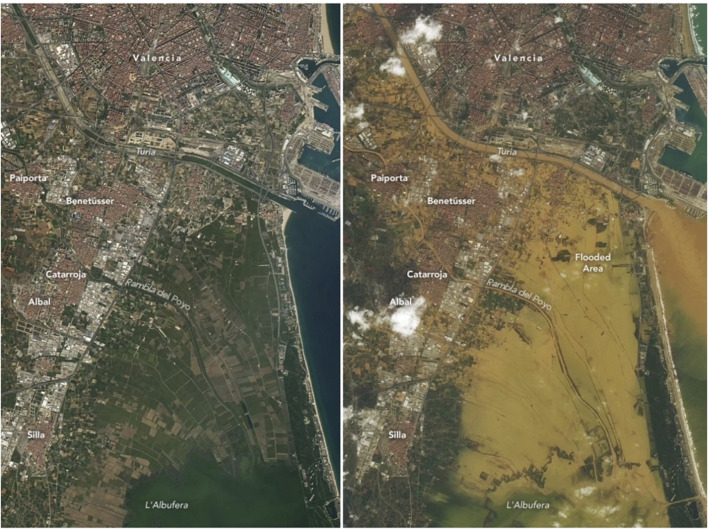
Images of the same geographic area in Valencia (Spain) taken at two different times in October 2024 by the U.S. satellite NASA-Landsat-8. The first image corresponds to Tuesday, October 8, while the second was taken on Wednesday, October 30, 1 day after the disaster (2024).

The images from Valencia by the U.S. Landsat-8 satellite reveal the severe impacts of the flood on 29 October 2024 [20] (see Figure 3). These images vividly showcase the dramatic transformation of the landscape before and after the heavy rainfall. The torrential rains—particularly intense at the river headwaters, where nearly 500 L per square meter were recorded in numerous locations—accelerated as they coursed through riverbeds and ravines, surging forward and devastated everything in their path.

A fourth aspect, particularly significant in Valencia, was the social and community impact, which was be driven by rising social tension, violence, and disputes over resources during this experience.

Job losses and economic hardships have become significant issues. The destruction of businesses and jobs has resulted in economic instability, further straining family and community dynamics and impacting the community's mental and physical health.

The damage of the educational disruption is severe and long lasting as was documented after the COVID-19 pandemic [21] school closures. Children have missed 2 months of schooling due to damaged schools, evacuations, or displacement, affecting their development and emotional wellbeing.

A fifth critical aspect was the rampant misinformation. During crises, the proliferation of misinformation, often termed “infodemics,” severely compromises the shared situational awareness (SSA) and impedes effective responses, which additionally fueled panic and hindered effective responses [22].

Finally, an additional disruptive factor was the political polarization, which generated immense frustration, led to loss of public trust and posed a significant obstacle to damage mitigation measures. The ruling party in the Valencian regional government represents a political faction in oppossition to that of the central government in Spain, leading to low levels of collaboration. This political discord not only caused delays in adequate prevention and responses, and beyond the delays, also fuelled public feelings of abandonment and frustration [23]. This aspect must be thoroughly examined to improve responses for future disasters and the need to build collective support across political divides.

### Recovery: Addressing Short-Term and Long-Term Risks and Community Resilience

Effective management of the immediate and long-term risks posed by the flood disaster requires coordinated and multifaceted interventions. The efforts to address the ‘wicked’ challenges posed by flood disasters provide valuable insights about the actions needed to mitigate future flood’s impacts:

#### Physical Health Risks

Preventive health measures play a critical role in reducing the risks of infectious diseases. Vaccination campaigns—specifically targeting tetanus and hepatitis A—and widespread distribution of hygiene kits, have proven effective in protecting public health [24]. Ongoing surveillance for potential outbreaks remains essential, particularly among vulnerable populations such as children, elderly, and those with preexisting conditions.

#### Mental Health Support

Rapid deployment of mental health professionals is needed to severely affected areas. Mental health professionals provided immediate counseling and crisis intervention and are needed to provide long term counseling and community-based support groups, crucial for fostering emotional recovery and resilience [25].

#### Community Recovery and Resilience

Rebuilding trust and resilience within affected communities is a key priority after disasters [26]. Post-disaster interventions should aim to increase social capital by strengthening family and community ties, and providing targeted financial assistance to affected households, increasing available social support and community connectedness.

### Financial and Social Planning for Future Challenges

The DANA floods in Valencia, highlighted several political, infrastructure and emergency response systems vulnerabilities. With a view to the future, the following lessons should be considered:

*Infrastructure Resilience:* Investments in better drainage systems, flood barriers, and resilient housing are critical to minimizing future risks.
*Climate Change Preparedness:* With extreme weather events becoming more frequent, Valencia must adopt climate-adaptive strategies to safeguard its population and the region.

Traditionally, economic analyses for investments in flood protection and prevention include the primary benefits such as damages averted, but there is a wide margin to analyze the secondary effects of these investments. Kahn et al. provide several examples of how investments in flood protection can create intended and unintended benefits, such as: job creation, new businesses, added recreational and green spaces with high participation of local contractors [27].

Cost-benefit analysis (CBA) can help in estimating the expected annual damages (EAD) if the intervention is not implemented. In addition, changes to land values within the floodplain or ecosystem rehabilitation are more challenging to estimate but should also be considered [28].

The secondary benefits of flood protection can be analysed through the triple dividends as proposed by Tanner [29]. This resilience framework approach considers three dimensions: avoiding losses during disasters (such as saving lives, reducing damages to infrastructure, and minimizing economic disruptions); unlocking economic potential, including increased investments, productivity, and land value due to reduced risk; and enhanced co-benefits, such as improved ecosystem services, transportation, and agricultural gains.

Standard disaster risk management (DRM) appraisals often overlook the second and third dividends, which provide benefits even in the absence of disasters. Policy decisions tend to underinvest in risk management due to visible upfront costs and uncertain, long-term returns. Integrating the triple dividend framework into resilience strategy assessments enhances transparency and supports more informed decision-making.

### Lessons Learned: Building Resilient Public Health Systems

The Valencia tragedy underscores the importance of a well-prepared policy, public health interventions, and robust communication plans for effective public health system resilience. Floods have substantial health impacts, both in the short and long term, and disproportionately affect vulnerable populations [30]. Protecting humans with a special focus on the elderly from the devastating effects of floods requires a comprehensive and dynamic risk management approach that includes prevention, preparedness, response and recovery, with collaboration across multiple sectors and disciplines.

## Conclusion

The devastating DANA flood of 29 October 2024, is a wake-up call for Valencia, the European Union and the world. It exposed the critical systems gaps in disaster prevention, preparedness, and response systems while offering invaluable lessons for the future. The key lessons include the need for:

Integrated disaster approaches: Public health should be reinforced by articulating and implementing actions for risk prevention and health protection from a precautionary perspective, and by implementing a *One Health* approach, addressing the interconnectedness of human, animal and environmental health. Measures aimed to reduce economic hardship and promote social cohesion are fundamental to long-term recovery. These include:

• Strengthening preparedness: Regular evaluations and updates of emergency protocols, coupled with international collaboration, could enhance the local ability to respond to future disasters. Preventive measures alone cannot eliminate flood risk. So public awareness, effective warning systems and ensuring resilient and responsive healthcare systems are also critical.
• Political cohesion and community engagement: Empowering communities to take an active role in their disaster preparedness, resiilence and recovery is key for building long-term resilience. Integrating the experiences of flood victims into community responses has been shown to be effective.
• Effective Communication: Supporting trusted and calm risk communications regarding evacuation routes, flood zones, self-protection activities and community responses can enable persons at risk to make informed decisions when time is critical.
